# Dual AAV gene therapy achieves recovery of hearing and auditory processing in a DFNB16 mouse model

**DOI:** 10.1002/ctm2.70571

**Published:** 2026-01-09

**Authors:** Sepideh Iranfar, Sophie Bagur, Chloé Felgerolle, Maxence Cornille, Najate Benamer, Hélène Le Ribeuz, Marie Giorgi, Sara Jamali, Amel Saoudi, Marie‐José Lecomte, Vincent Michel, Brice Bathellier, Saaid Safieddine

**Affiliations:** ^1^ Université Paris Cité, Institut Pasteur, AP‐HP, INSERM, CNRS, Fondation Pour l'Audition, Institut de l'Audition IHU reConnect Paris France; ^2^ Ecole Doctorale Physiologie, Physiopathologie et Thérapeutique Sorbonne Université Paris France; ^3^ Institut Pasteur INSERM Pathogenesis of Vascular Infections Unit Paris France

**Keywords:** AAV, DFNB16, gene therapy, hearing, sound processing, stereocilin

## Abstract

**Background:**

DFNB16, the second most common genetic cause of hearing loss, is caused by mutations of the *STRC* gene encoding stereocilin, a protein essential for the effective functioning of outer hair cells (OHCs) as cochlear amplifiers. *Strc* ^−/−^ mice, which lack stereocilin, display severe to profound deafness and constitute a relevant preclinical model for DFNB16.

**Methods:**

Using Strc^−^/^−^ mice, we developed a gene therapy strategy based on the use of dual AAV9‐PHP.eB vectors to deliver the full‐length *Strc* cDNA. Therapeutic efficacy was assessed by evaluating stereocilin expression, OHC bundle architecture, and their attachment to the tectorial membrane, together with functional recovery using distortion product otoacoustic emissions (DPOAEs), auditory brainstem responses (ABR) measurements and Go/No‐Go behavioral testing with psychometric analysis.

**Results:**

Dual‐AAV–mediated Strc gene delivery restored stereocilin expression, OHC bundle architecture and their attachment to the tectorial membrane, leading to the recovery of cochlear amplification and hearing to near normal thresholds, as confirmed by distortion product otoacoustic emission (DPOAE) and auditory brainstem response measurements. Behavioural assessment showed that treated *Strc* ^−/−^ mice regained normal frequency discrimination, indicating a restoration of higher‐order auditory processing, up to 100 days post‐treatment.

**Conclusion:**

These findings provide the first proof‐of‐principle that peripheral gene therapy can restore OHC function, cochlear amplification and central auditory perception in a DFNB16 model.

**Key points:**

- Dual AAV‐mediated gene delivery restored peripheral hearing in a DFNB16 preclinical mouse model.
- The same treatment also restored central auditory processing.
- AAV‐mediated gene therapy represents a promising curative strategy for DFNB16.
- These results reinforce the translational potential for treating human genetic deafness.

## Graphical abstract

DFNB16 is among the most prevalent forms of congenital deafness, caused by mutations in the *Stereocilin* gene. Although no treatment currently exists, gene therapy represents a promising curative approach. Here, we demonstrate that AAV‐mediated gene delivery in a DFNB16 mouse model restored both peripheral hearing and central auditory processing.

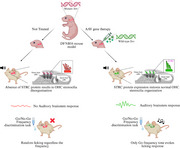

## 1 INTRODUCTION

Deafness is one of the main causes of human disability worldwide, affecting more than 5% of the world population. An estimated one in 700 children is born with congenital deafness, with genetic factors accounting for about 80% of cases.^1^ To date, more than 200 genes have been associated with deafness according to the Deafness Variation Database, including 156 genes linked to nonsyndromic hereditary hearing loss.^2^, ^3^ Cochlear implants and hearing aids are currently the only options available for managing sensorineural hearing loss in affected patients. However, with these devices, auditory function and speech perception in noisy environments remain significantly impaired,^4^, ^5^, ^6^ increasing the risk of social isolation and dementia in adult patients.^7^ The high prevalence of genetic deafness in humans has driven pioneering preclinical and clinical studies aimed at treating some inherited hearing loss with gene therapy, primarily using adeno‐associated virus (AAV) vectors for gene transfer.^1^ Encouraging data have recently been obtained in mouse models of DFNB1 using gene therapy targeting connexin 26, the most common cause of genetic hearing loss.^8^, ^9^ Remarkably, proof‐of‐concept has been established in mouse models of DFNB9, a congenital form of deafness caused by OTOF mutations, and clinical trials for DFNB9 gene therapy have already confirmed the therapeutic potential of this approach for genetic forms of human hearing loss.^10^, ^11^, ^12^, ^13^, ^14^, ^15^

In this study, we evaluated the efficacy of gene therapy in a mouse model of DFNB16, the second most prevalent type of congenital hearing loss.^16^, ^17^, ^18^ DFNB16 is a recessive form of mild‐to‐moderate human deafness caused by mutations of the stereocilin (*STRC*) gene, accounting for approximately 11% of all reported cases.^19^, ^20^ In addition, the carrier frequency of pathogenic STRC variants in normal‐hearing individuals has been estimated at 1.6–1.8%.^21^, ^22^ There is currently no curative treatment for DFNB16. Solutions remain limited to rehabilitation approaches, which, while effective for improving the amplification or transmission of sound, do not restore the essential features of sound processing underlying natural hearing.^23^

We used a stereocilin‐deficient mouse line (*Strc* ^−/−^) with the key characteristics observed in human DFNB16 deafness to model this condition. In wild‐type mice, the STRC protein is found at the tips of the outer hair cells (OHC) stereocilia, which function as cochlear amplifiers, enhancing the frequency selectivity of the hearing organ.^24^ In *Strc^−^* ^/−^ mice, the OHCs lack the upper horizontal connectors linking adjacent stereocilia both between and within the three rows, and the attachment crowns responsible for anchoring the tallest row of stereocilia to the tectorial membrane.^25^ This structural abnormality results in a disorganisation of the stereocilia, impairing the ability of OHCs to act as frequency‐selective amplifiers. Consequently, *Strc* ^−/−^ mice experience mild to profound hearing loss.^22^, ^24^ In this study, we developed an AAV‐mediated gene therapy targeting OHCs to restore cochlear function and central auditory signal processing in a preclinical model of DFNB16 deafness. Because the *Strc* cDNA exceeds the capacity of a single AAV, and guided by translational considerations and the promising outcomes of ongoing DFNB9 clinical trials,^11^, ^13^, ^14^, ^15^ we adopted a dual‐AAV strategy closely reproducing the one applied in DFNB9.^10^ We evaluated its efficacy using DPOAEs and auditory brainstem responses (ABRs), widely recognised as standard indicators of peripheral auditory function that respectively assess OHC activity and synchronised neural responses from the auditory nerve to the brainstem.^26^, ^27^ In parallel, we sought to define the optimal therapeutic window for intervention, given that the timing of gene delivery may critically influence both the extent and durability of hearing recovery. Since ABR measurements alone do not fully capture auditory perception and higher‐order processing, we conducted behavioural assessments involving a frequency discrimination task based on a Go/No‐Go paradigm.^28^, ^29^, ^30^, ^31^ This approach enabled us to demonstrate the perceptual relevance of the intervention by probing auditory‐driven decision‐making and fine frequency discrimination in treated animals.

## 2 RESULTS

### 2.1 *Strc^−/−^* mice develop severe to profound deafness

**FIGURE 1:**
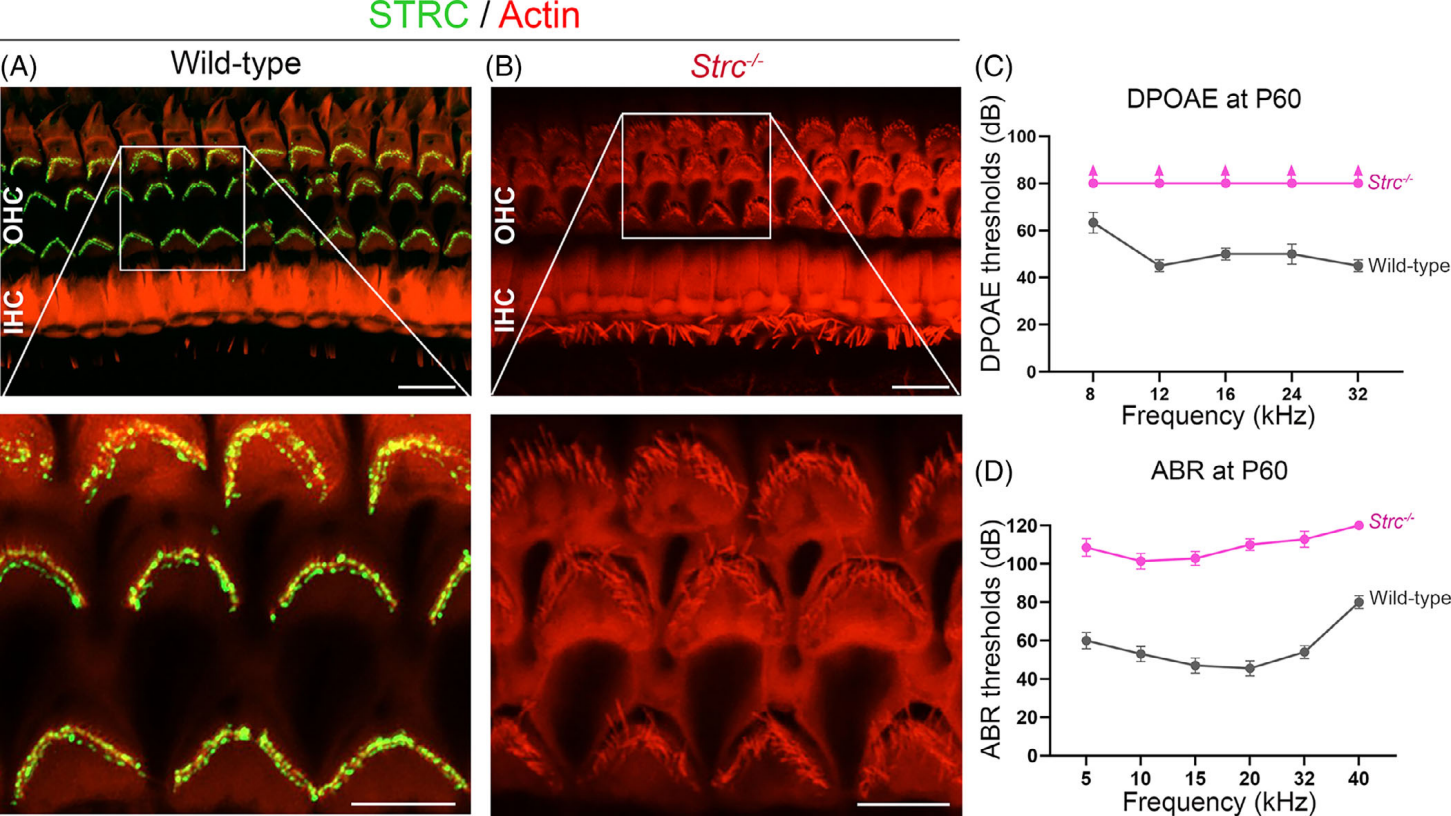
Strc^−/−^ mice experience hearing loss due to a severe disorganisation of outer hair cell stereocilia. Confocal images of the organ of Corti from the middle turn of the cochleas of a wild‐type mouse (A) and a Strc^−/−^ mouse (B) stained for STRC (in green) and actin (in red) at P60, focusing on the stereocilia of inner (IHCs) and outer (OHCs) hair cells. Insets show higher magnifications of the boxed areas, highlighting STRC localisation at the tips of OHC stereocilia in wild‐type cochlea, in which the OHC bundles have a characteristic V‐shaped arrangement. By contrast, Strc^−/−^ OHC stereocilia, which lack STRC, have a disorganised bundle architecture. Scale bars, 10 and 5 µm for overviews and insets, respectively. (C) DPOAE (2*f*_1_–*f*_2_) thresholds obtained for the wild‐type (grey) and Strc^−/−^ (pink) mice at P60 (mean ± SEM, *n* = 10). (D) ABR thresholds recorded in wild‐type (grey) and Strc^−/−^ (pink) mice at P60 (mean ± SEM, *n* = 10).

**FIGURE 2:**
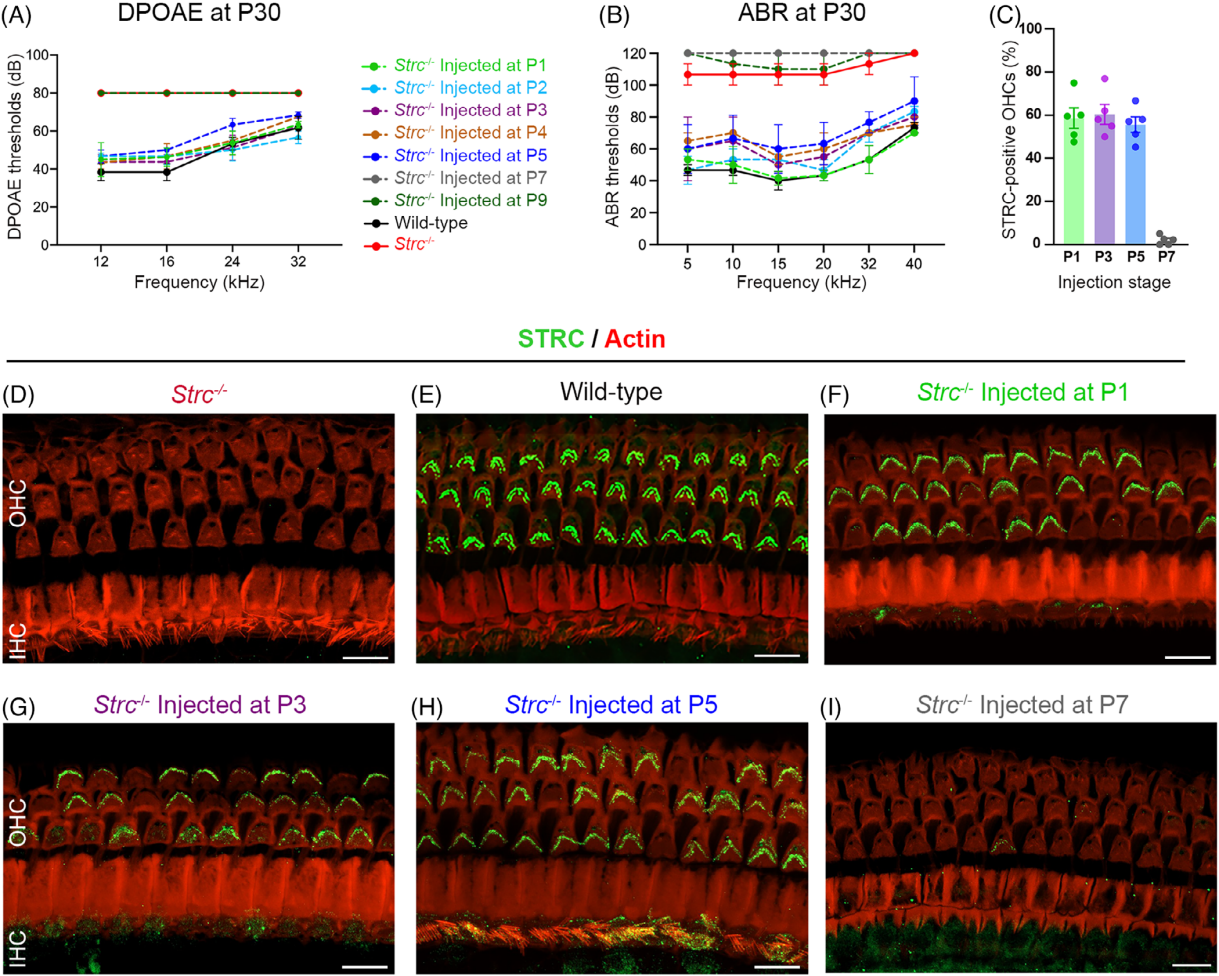
Restoration of STRC expression and hearing function in *Strc* ^−/−^ mice by dual AAV gene therapy during cochlear maturation. (A) DPOAE and ABR (B) recordings at P30 from *Strc* ^−/−^ mice treated with the therapeutic vector at different post‐natal ages (colour‐coded) show that hearing restoration is achieved when treatment is administered up to P5 (mean ± SEM, *n* = 3), (C) Histogram showing OHC transduction efficiency in *Strc* ^−/−^ mice following dual AAV delivery at different post‐natal ages. (D–F) Confocal images of the organ of Corti from the middle turn of *Strc* ^−/−^ mouse (D) and wild‐type mouse (E), as well as from *Strc* ^−/−^ cochleas injected at P1 (F), P3 (G), P5 (H) and P7 (I), immunostained 4‐weeks post‐injection for STRC (green) and actin (red). Robust STRC expression in OHCs, comparable to wild‐type, was observed when treatment was delivered up to P5. The OHCs expressing therapeutic STRC displayed stereociliary bundle organisation and cohesion comparable to that of wild‐type mice. Scale bars, 10 µm.

The *Strc* ^tm1Ugds/tm1Ugds^, referred to as *Strc* ^−/−^ mouse, was previously engineered and characterised by Verpy et al.,^24^, ^25^ and its features were further confirmed here. The organ of Corti of these mice lacks STRC protein, which is expressed and localised mainly at the tips of the OHC stereocilia in wild‐type mice (Figures 1A and 2E). The absence of STRC in *Strc* ^−/−^ mice severely disrupts OHC bundle cohesion, resulting in a disorganised stereociliary architecture (Figures 1B and 2D,I). This structural disruption causes detachment of the hair bundles from the tectorial membrane and impairs the cochlear amplifier function of OHCs, leading to elevated hearing thresholds, as evidenced by elevated thresholds in both ABR and DPOAE recordings (Figure 1C,D). Longitudinal auditory assessments showed that *Strc* ^−/−^ mice display congenital progressive hearing loss, culminating in profound deafness by post‐natal day 30 (P30) (Figure 2A,B).

### 2.2 Design of the dual AAV therapeutic vector

Because the murine *Strc* coding sequence (5430 bp) exceeds the ∼4700 bp packaging limit of a single AAV, and to maximise translational relevance for DFNB16 gene therapy, we adopted the dual‐AAV strategy previously used to restore hearing in the DFNB9 mouse model,^10^ which has since formed the basis of several clinical trials.^11^, ^13^, ^14^, ^15^ The *Strc* cDNA (NM_080459.2) was split at a site that enabled both expression cassettes to remain within the AAV packaging limit, share a highly recombinogenic exogenous sequence, and preserve a native *Strc* splice site. We selected AAV9‐PHP.eB based on our recent comparative analysis of AAV2, AAV8, AAV9‐PHP.eB and Anc80L65 across embryonic, neonatal and adult stages, which revealed its clear superiority in transducing OHCs.^32^, ^33^ Specifically, the AAV‐*Strc* N‐terminal (NT) recombinant vector carries the promoter sequence, the 5′ portion of the cDNA (*Strc*‐NT, nucleotides 1–2775), and a splice donor site, whereas the AAV‐*Strc* C‐terminal (CT) recombinant vector contains a splice acceptor site followed by the 3′ portion of the cDNA (*Strc*‐CT, nucleotides 2776–5430) (Figure S1A). We evaluated the reconstitution of the full‐length STRC protein by transfecting HeLa cells with either the *Strc*‐NT or *Strc*‐CT, or with both plasmids in combination. Immunostaining 48 h post‐transfection showed that STRC was present only in cells cotransfected with both constructs, indicating successful recombination and splicing (Figure S1B). In addition, RT‐PCR analysis carried out on RNA extracted from transfected cells, targeting a ∼1 kb region encompassing the NT–CT junction, followed by Sanger sequencing of the purified amplicon, confirmed accurate splicing and alignment with the native *Strc* sequence at the exon 8–exon 9 junction (Figure S1C,D). Reliable western blot analysis of protein extracts from cells co‐transfected with the dual *Strc* plasmids, using an anti‐STRC antibody, revealed a distinct band at the expected molecular weight (∼194 kDa), demonstrating successful plasmid DNAs recombination, splicing and expression of the full‐length STRC protein. No STRC immunoreactivity was detected in extracts from cells transfected with either *Strc*‐NT or *Strc*‐CT plasmid alone, nor in non‐transfected cells (Figure S1E).

### 2.3 Therapeutic time window in the DFNB16 mouse model

We first sought to verify in vivo the specificity of dual AAV‐mediated *STRC* expression described above. To this end, each vector was administered either alone or in combination via the round window membrane (RWM) in *Strc* ^−/−^ mice. The organ of Corti from injected cochleas was microdissected and immunostained for stereocilin. STRC protein was detected exclusively in the organ of Corti from cochleas that received both vectors and was correctly localised at the tips of OHC stereocilia, mirroring the distribution of native STRC, and thereby validating our in vitro findings (Figure S1F).

We next investigated the optimal developmental window for therapeutic efficacy by injecting 1 µL of dual *Strc* vector into the cochlea of *Strc^−/−^* mice through the RWM at various post‐natal stages, specifically at P1, P2, P3, P4, P5, P7, P9 and P14–18 (at least *n *= 3 mice injected at each time point). We first assessed the restoration of cochlear amplification and hearing thresholds 4 weeks post‐injection by recording DPOAE and ABR. Dual‐vector administration up to P5 successfully restored both DPOAE and ABR thresholds to levels near those of wild‐type controls (*p* > .01, two‐way ANOVA). By contrast, injections performed after P5 failed to rescue DPOAE or restore hearing thresholds (*p* < .0001 relative to the wild‐type group, two‐way ANOVA; Figure 2A,B). We then assessed the restoration of STRC protein expression in *Strc* ^−/−^ mouse cochleas 4 weeks post‐injection. The cochleas were fixed and the organs of Corti were microdissected and immunolabelled for STRC and actin. Dual AAV‐mediated delivery of fragmented *Strc* cDNA at time points up to P5 resulted in robust stereocilin expression and normal targeting to tips of OHCs stereocilia, yielding a pattern similar to that of wild‐type mice. However, in *Strc* ^−/−^ mice treated after P5, OHC transduction efficiency dropped sharply to less than 5%, and most of OHCs lacking stereocilin exhibited abnormal stereocilia bundle morphology, similar to that observed in untreated *Strc* ^−/−^ mice (Figure 2D–I). This finding mirrors the absence of hearing restoration observed when treatment was administered at these later developmental stages. It also aligns with previous reports using several AAV serotypes expressing GFP, including AAV9‐PHP.eB, which exhibited markedly reduced OHC transduction efficiency in more mature cochlea.^32^, ^33^ Together with the need for efficient coinfection inherent to the dual‐vector strategy, this likely accounts for the lack of therapeutic efficacy and highlights the link between hair cell transduction efficiency and hearing recovery. These results also suggest that successful treatment in adult *Strc^−/−^* mice might be achievable with a more efficient serotype specifically targeting OHCs at the mature stage.

### 2.4 Gene therapy rescues STRC expression and targeting, along with the structural integrity of OHC bundles

**FIGURE 3:**
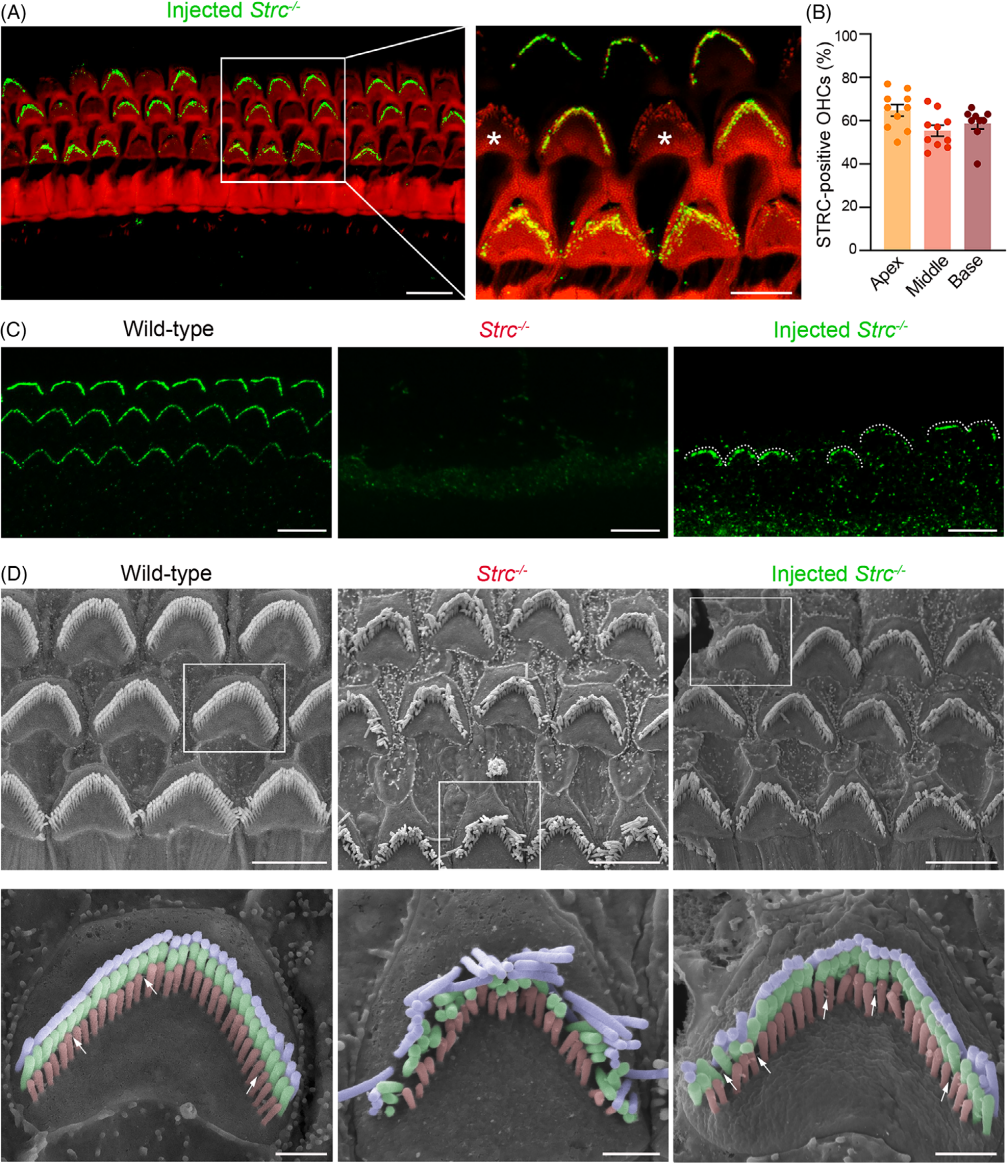
Gene therapy restores STRC expression and localisation, rescuing OHC bundle morphology in *Strc^−/−^* mice. (A) Confocal image of the organ of Corti from the apical region of a *Strc^−/−^* mouse cochlea treated at P2, immunostained at P60 for STRC (green) and actin (red). The inset shows a higher magnification of the boxed region, demonstrating that gene therapy restores STRC expression and correct targeting to OHC stereocilia tips (asterisks indicate the non‐transduced OHCs with disorganised hair bundles). Scale bars: 10 µm (main image), 5 µm (inset). (B) Histogram showing OHC transduction rate. Data presented in mean ±  SEM, *n* = 10. (C) Confocal images of the tectorial membrane from wild‐type (left), *Strc* ^−/−^ (middle) and treated *Strc* ^−/−^ (right) mice stained for STRC (green) at P30. STRC‐immunoreactive imprints on the lower surface of the tectorial membrane (dashed line) are restored in the treated *Strc* ^−/−^ mice. Scale bars: 5 µm. (D) Low‐ and high‐magnification scanning electron micrographs (with false‐colour hair bundles) of the apical region of the mouse organs of Corti, showing OHC bundle architecture in wild‐type (left), *Strc* ^−/−^ (middle) and treated *Strc* ^−/−^ (right) mice at P30. The OHC bundles of injected *Strc* ^−/−^ mice display a morphology similar to those of wild‐type, owing to the restoration of the horizontal top connectors (arrows). Scale bars, 10 and 1 µm for overviews and insets, respectively.

We focused on the neonatal stages up to P2, as administration of the dual *Strc* vector at this time point yielded the most robust restoration of auditory thresholds in *Strc* ^−^/^−^ mice. At 4 and 8 weeks post‐injection (i.e., P30 and P60), two stages reflecting distinct degrees of stereocilia disorganisation in untreated *Strc* ^−/−^ mice, cochleas were collected, processed for immunohistochemistry, and analysed using a confocal microscope. STRC protein re‐expression was detected in the treated cochleas, with correct localisation at the tips of OHC stereocilia (Figures 2F and 3A). STRC‐positive stereocilia had a characteristic V‐shaped bundle morphology with aligned tips, resembling the wild‐type structure and contrasting with the disorganised bundles observed in *Strc* ^−/−^ controls (Figures 3A and S2). Quantitative analysis showed that 64.2 ± 2% of apical, 55.4 ± 2% of medial, and 58.7 ± 3% of basal OHCs were STRC‐positive and had a cohesive bundle architecture (Figure 3B; mean ± SEM, *n* = 10).

It is noteworthy that the dual‐AAV9‐PHP.eB approach achieved ∼60% transduction efficiency, compared with nearly 100% using a GFP reporter.^32^, ^33^ This likely reflects post‐transcriptional and protein‐processing constraints, together with the need for (i) co‐infection of OHCs by both vectors and (ii) accurate recombination of the gene segments within these cells. Remarkably, the tectorial membrane of treated *Strc* ^−/−^ mice immunolabelled for stereocilin at P30 exhibited V‐shaped stereocilin imprints on its lower surface, closely resembling those seen in wild‐type mice (Figure 3C). This finding indicates that therapeutically expressed STRC was properly trafficked to the tallest stereocilia, successfully restoring the crown attachments of the tectorial membrane. Scanning electron microscopy analysis further confirmed the preservation of stereocilia bundle cohesion in OHCs of the treated cochlea with 49 ± 5% preservation (mean ± SEM, *n* = 3), and visible links between adjacent stereocilia at P30 (Figure 3D). Notably, the horizontal top connectors essential for bundle integrity were present in treated mice but absent in untreated controls (Figure 3D, insets). Together, these results demonstrate that dual viral delivery of the *Strc* cDNA not only restores STRC expression and its correct localisation within OHC bundles but also enables the protein fulfil its structural role as a connector,^25^ thereby preventing bundle degeneration by restoring stereociliary links and maintaining bundle cohesion in treated *Strc* ^−/−^ mice.

### 2.5 Gene therapy restores cochlear amplification and improves hearing in *Strc* ^−/−^ mice

**FIGURE 4:**
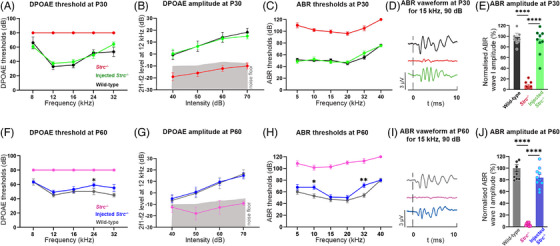
Gene transfer durably restores cochlear amplifier function and ABR thresholds in *Strc^−/−^* mice. The longevity of restored hearing after gene therapy administered at P2 was assessed by DPOAEs and ABRs recording at P30 (A–E) and P60 (F–J). At both time points, treated *Strc* ^−/−^ mice exhibited restoration of DPOAE thresholds (A and F) and amplitudes (B and G), comparable to those of wild‐type mice (shaded area indicates the noise floor). Similarly, ABR thresholds (C and H) were rescued to near‐normal levels, except for slightly but significantly elevated thresholds at 10 and 32 kHz at P60. Representative ABR waveforms recorded at 15 kHz and 90 dB SPL (D and I) used to evaluate wave I amplitudes (E and J), which were restored to wild‐type levels at both time points. Data present mean ± SEM, *n *= 10 per group, *p < .05, **p < .01, ****p < .0001, two‐way ANOVA.

We assessed the functional impact of STRC re‐expression and normal targeting within the OHCs by recording DPOAE, a reliable indicator of OHC‐mediated sound amplification. In the treated *Strc* ^−/−^ mice, robust DPOAE responses well above the background noise were recorded at the intermodulation frequency 2*f*_1_–*f*_2_ in response to two pure tones referred to as *f*_1_ and *f*_2_, whereas no DPOAE were elicited in untreated *Strc* ^−/−^ mice. We further analysed DPOAE thresholds and amplitudes at 12 kHz, corresponding to a region of the cochlea in which AAV‐mediated gene therapy achieves a high transduction efficiency. Measurements were performed in both treated *Strc* ^−/−^ mice and wild‐type controls at P30 and P60. In treated *Strc* ^−/−^ mice, DPOAE thresholds and amplitudes were similar to wild‐type levels at P30 and P60 (Figure 4A,B,F,G) (for thresholds at P30 and P60: *p *= .75 and, *p *= .14, two‐way ANOVA, *n *= 10 and 5 respectively; for amplitudes at P30 and P60: *p =* .92 and *p =* .98, two‐way ANOVA, *n *= 10 and 5, respectively), indicating effective restoration of OHCs as cochlear amplifier function.

Treated *Strc* ^−/−^ mice showed robust improvements across the 5–40 kHz range, reaching near wild‐type levels, with only minor threshold elevations at 10 and 32 kHz at P60 (Figure 4C,H). The mean hearing threshold across 5–40 kHz was 55.6 ± 4.6 dB at P30 (versus 54.7 ± 4.4 dB in wild‐type mice, *p *= .92, two‐way ANOVA, *n* = 10; Figure 4C), 64.6 ± 4.8 dB at P60 (versus 56.5 ± 5.1 dB in wild‐type mice, *p* = .90, two‐way ANOVA, *n* = 10; Figure 4H), and 66.9 ± 5.1 dB at P90 (versus 59.3 ± 5.4 dB recorded in wild‐type mice, *p* = .57, two‐way ANOVA, *n* = 6; Figure S3) indicating no statistically significant difference from wild‐type mice at any age, despite a modest increasing trend at later stages.

Furthermore, the amplitude of ABR wave I, reflecting the synchronised firing of primary auditory neurons in response to acoustic stimuli consisting of 15 kHz tone bursts at 90 dB, was almost of the same level in treated *Strc* ^−/−^ mice and wild‐type mice, demonstrating a restoration of cochlear gain in the treated mice (at P30 and P60: *p* = .83 and *p* = .18, one‐way ANOVA, *n *= 10 and 7, respectively; Figure 4D,E,I,J). The restored hearing thresholds remained stable for up to 90 days following a single administration of gene therapy in the treated mice (Figure S3).

### 2.6 Gene therapy restores frequency discrimination

**FIGURE 5:**
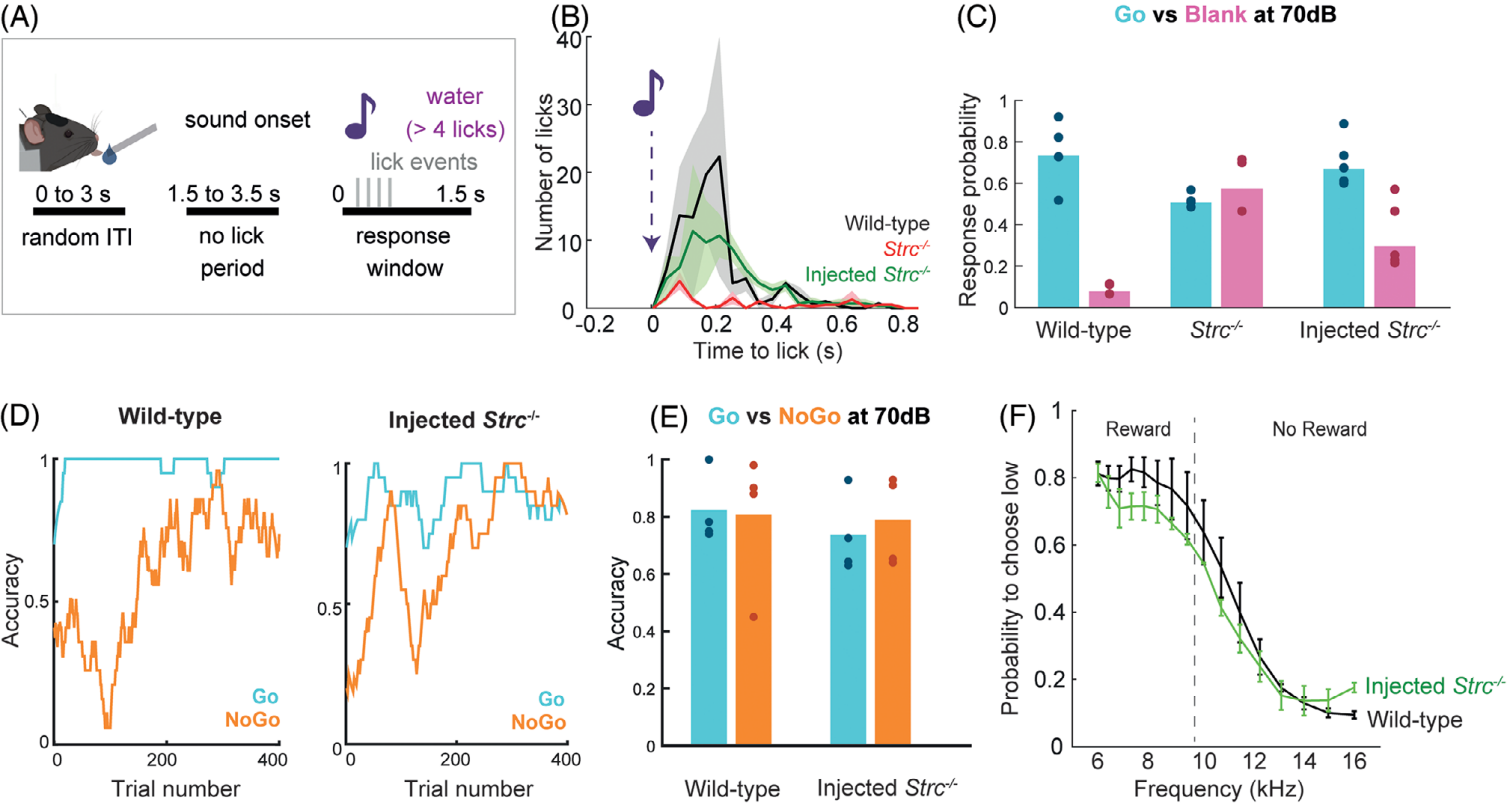
Gene therapy restores auditory‐driven behaviour and frequency discrimination in treated *Strc^−/−^* mice assessed from P70 to P100 following P2 administration. (A) Schematic representation of a head‐fixed mouse during the frequency discrimination task in which water‐deprived mice are trained to lick four times within a 1.5‐s period after the onset of a Go‐stimulus to receive a water reward. The random ITI (inter‐trial interval varies between 0 and 3 s). (B) Graph showing the mean ± SEM of the number of licks following Go‐stimulus onset in wild‐type (black), untreated *Strc^−/−^* (red) and treated *Strc^−/−^* (green) mice (*n* = 4 per group). (C) Response probability during Go trials (70 dB pure tone, blue) and blank trials (no sound, pink). Treated *Strc* ^−/−^ mice exhibited a high response probability in Go trials, similar to wild‐type mice, whereas untreated *Strc* ^−/−^ mice responded at randomly in both trial types (*n* = 4 per group). (D) Representative learning curves showing accuracy over 500 trials for Go and No‐Go trials in wild‐type and treated *Strc^−/−^* mice, demonstrating acquisition of the auditory‐driven task. (E) Discrimination accuracy between Go (rewarded) and No‐Go (non‐rewarded) stimuli in wild‐type and treated *Strc^−/−^* mice, showing that treated *Strc^−/−^* mice correctly distinguish between low and high frequencies, as in wild‐type controls (*n* = 4 per group). (F) Psychometric measurements for wild‐type (black) and treated *Strc* ^−/−^ (green) mice, showing similar psychometric curves for these two groups of mice (*n* = 4 per group, dashed line indicates the threshold frequency: 9.8 kHz, licking at <9.8 kHz is rewarded, and at >9.8 kHz is not rewarded).

OHC amplification enhances frequency selectivity, which is crucial for speech intelligibility.^23^, ^28^ Frequency‐encoded signals travel from the cochlea to the auditory cortex via the brainstem and medial geniculate body, enabling precise frequency discrimination.^4^ In this study, we evaluated the accuracy of frequency discrimination, which is impaired in DFNB16 due to the absence of a functional cochlear amplifier.^4^ We evaluated sound detection and response in *Strc* ^−/−^, treated *Strc* ^−/−^ and unilaterally deaf wild‐type mice. Water‐deprived mice received a reward for licking within a 1.5 s window after hearing a 500 ms pure tone (6 or 16 kHz, 70 dB; Figures 5A and S4). Wild‐type mice quickly learned to lick in response to the tone and to refrain from licking during control blank trials in which no sound was delivered (Figure 5B,C). By contrast, *Strc* ^−/−^ mice displayed no clear licking pattern, responding randomly during both sound and blank trials, suggesting an inability to perceive the tone. However, treated *Strc* ^−/−^ mice responded selectively to the sound, clearly distinguishing it from blank trials (Figure 5B,C). Thus, the treated mice not only detected the sound but also integrated the sensory input to guide learning‐driven behaviour. We then trained the treated *Strc* ^−/−^ and wild‐type mice to perform a Go/No‐Go discrimination task in which they were rewarded for responding to the Go (e.g., 6 kHz) stimulus and punished by a time‐out period for responding to the No‐Go stimulus (e.g., 16 kHz). In this task, the treated *Strc* ^−/−^ mice discriminated between the high‐ and low‐frequency sounds with an accuracy of more than 80%, like their wild‐type counterparts (Figure 5D,E). Following on the learning of this discrimination task, mice were introduced to a psychometric task in which they were required to categorise 16 logarithmically scaled sounds into low‐ and high‐frequency categories (threshold frequency: 9.8 kHz). Interestingly, the psychometric curves obtained for the treated *Strc* ^−/−^ mice were very similar to those for wild‐type mice at P100. We detected no change in the slope of the psychometric curves; this slope quantifies fine differentiation between sound frequencies (Figure 5F). Thus, the efficacy of peripheral gene therapy extends beyond the simple restoration of cochlear function to re‐establishment of normal sound signal processing via the central auditory pathway to drive sensory‐motor learning.

## 3 DISCUSSION

The aim of this study was to develop a durable and effective gene therapy for DFNB16, the second most common genetic cause of deafness in humans, caused by mutations of the *STRC* gene.^16^, ^17^, ^18^ To this end, we used *Strc* ^−/−^ mice, a well‐established preclinical model for DFNB16.^24^, ^25^ Because the *Strc* cDNA is too large to fit within a single AAV capsid, we employed a dual‐AAV delivery strategy previously established for DFNB9 gene therapy, which has served as the basis for several ongoing clinical trials with encouraging results.^11^, ^13^, ^14^, ^15^ By doing so, we achieved strong STRC expression, accurately localised at the tips of OHC stereocilia, which displayed a clear V‐shaped morphology, in approximately 60% of cells, closely matching the transduction efficiency obtained with intein‐based dual‐AAV gene therapy.^22^ Furthermore, scanning electron microscopy analysis revealed that gene therapy rescued the horizontal top connectors of the stereocilia, thereby re‐establishing the cohesiveness of the OHC stereocilia bundles and the attachment crowns to tectorial membrane, both of which are essential for effective cochlear amplification.^24^ Functional recovery was confirmed by the recording of DPOAE and auditory ABR, which showed that treated *Strc* ^−/−^ mice had hearing thresholds similar to those of wild‐type controls across all tested frequencies. However, while ABR recordings are a valuable tool for assessing sound‐evoked neural activity along the auditory nerve and brainstem, they provide limited insight into higher‐order auditory processing and perceptual outcomes, the critical determinants of real‐world hearing performance.^34^, ^35^ We therefore assessed whether restoration of OHC function through gene therapy resulted in perceptually meaningful recovery by evaluating auditory‐driven behaviour in a Go/No‐Go task designed to probe frequency discrimination.^28^, ^29^, ^30^, ^31^ In this paradigm, untreated *Strc* ^−/−^ mice failed to learn the task, consistent with their profound auditory deficits. By contrast, treated *Strc* ^−/−^ mice successfully learned to respond to a target frequency and to ignore non‐target tones, achieving performance levels comparable to those of wild‐type controls. Psychometric curve analyses revealed similar thresholds and slopes in the treated and wild‐type groups, indicating not only a restoration of hearing sensitivity, but also rescue of fine frequency resolution essential for perceptual discrimination. Although our dual‐AAV therapy was specifically designed to restore *STRC* expression in OHCs, the treated mice also exhibited normalisation of central auditory processing, indicating that the auditory pathway remained functionally operational. The recovery of frequency discrimination therefore reflects improved cochlear amplification and enhanced signal fidelity transmitted to the central auditory system, which in turn processes frequency information normally. In doing so, this work bridges a key gap between cochlear recovery and central auditory processing, an aspect often overlooked in previous preclinical studies.^10^, ^22^, ^33^ Such functional validation is essential for clinical translation, in which perceptual and behavioural outcomes are key measures of therapeutic success. Together, these findings demonstrate the restoration of cochlear function through gene therapy is sufficient to reactivate central auditory pathways, underscoring the intrinsic plasticity of the auditory system and the translational potential of gene therapy for DFNB16 deafness. This level of functional recovery was achieved with the transduction of approximately 60% of OHCs. This suggests that either STRC levels in some OHCs were below the threshold for detection with antibodies or that intercellular compensation occurred, with functional OHCs partially compensating for the lack of activity of their non‐transduced neighbouring cells. Importantly, these findings suggest that full restoration of hearing thresholds and auditory perception to normal in DFNB16 mouse models may be achievable using a recombinant vector optimised to increase transduction rates and ensure robust stereocilin expression in OHCs. This conclusion is further supported by our previously published results, which demonstrate a clear correlation between the transduction rate of inner ear sensory cells and the degree of functional recovery, both vestibular and cochlear.^36^, ^37^ Moreover, it is important to note that DFNB16 patients using hearing aids often face limitations, including poor sound quality, a lack of efficacy in noisy environments and only partial benefits.^23^, ^38^, ^39^ Here, we provide the first proof‐of‐principle that peripheral gene therapy can durably restore OHC function, cochlear amplification and key aspects of central auditory processing in a DFNB16 mouse model. Given the structural and functional similarities between the murine and human cochleas, we anticipate that comparable therapeutic outcomes could be achieved in DFNB16 patients through the use of a suitably optimised AAV serotype capable of targeting human OHCs.

## 4 MATERIALS AND METHODS

### 4.1 *Strc^−/−^* mice

The animals were housed in the animal facility of the *Institut de l'Audition* of *Institut Pasteur*. All procedures were approved by the ethics committees of the *Université Paris Cité*, INSERM, and *Institut Pasteur*. The *Strc* ^−/−^ mice (*Strc^tm1Ugds/tm1Ugds^*, stereocilin knockout) have been described in detail elsewhere.^24^ Briefly, The *Strc* ^−/−^ mice were generated using the Cre‐lox system via a cross between 129/Sv (transgenic mice containing floxed regions of exons 2 and 3 in *Strc* gene) and C57BL6/J (transgenic mice expressing PGK‐Cre recombinase early and ubiquitously) mice, resulting in the deletion of exons 2 and 3 of the *Strc* gene. This deletion caused 30 amino acids to be out‐of‐frame, resulting in an incomplete signal peptide and a non‐functional STRC protein. The *Strc* ^−/+^ and *Strc* ^+/+^ mice had no auditory deficits and were used as wild‐type controls with similar genetic backgrounds.

### 4.2 Recombinant vector constructs and packaging

The full‐length coding sequence of the murine stereocilin cDNA (NM_080459.2) was split into two parts, a 5′ part (*Strc‐*NT, nucleotides 1–2775) and a 3′ part (*Strc‐*CT, nucleotides 2776–5430), each carrying an alkaline phosphatase recombinogenic bridging sequence (AP). The NT fragment included the P0101‐smCBA‐*Strc*‐NT‐splice donor (SD) site, and the CT fragment contained the P0101‐*Strc‐*CT‐splice acceptor (SA) site (GenScript). All vectors were packaged into AAV9‐PHP.eB capsids at the viral vector facility of the University of Zurich at a titer of 1.8 × 10^13^ gc/mL for NT and 3.3 × 10^13^ gc/mL for CT fragments. We used a dilution of 2:1 of *Strc*‐NT and *Strc‐*CT for dual AAV preparation, and a volume of 1 µL of the dual preparation was delivered into the mouse cochlea.

### 4.3 Viral vector delivery

The intracochlear injections were performed as previously described.^36^ Our surgical procedure involved the use of isoflurane‐induced anaesthesia (4% for induction and 1.5% for maintenance). Local anaesthesia with lidocaine (5 mg/kg) was applied at the surgical site and the skin was disinfected with betadine. A retro‐auricular incision was made, and the subcutaneous layers and muscles were slightly dissected to access the otic bulla and identify the RWM. The viral vector was slowly injected through the RWM (100 nL/s) with a nanolitre injector (World Precision Instruments) coupled to a glass micropipette (10 µm diameter). The RWM was rapidly sealed with a small plug of muscle and the incision was closed with a drop of biological glue (Vetbond 3M).

### 4.4 Auditory brainstem responses

The mice were anaesthetised by intraperitoneal injection of a mixture of 10% ketamine (Imalgene^®^; 100 mg/kg) and 2% xylazine (Rompun^®^; 20 mg/kg) and were placed individually on a homeothermic pad to maintain body temperature, within a sound‐attenuating chamber. Three electrodes – the ground, reference and recording electrodes – were used to record ABR over the 5–40 kHz frequency range, at decreasing intensities, from 110 to 10 dB. The hearing threshold was defined as the lowest stimulus evoking a detectable brainstem response. The amplitude of wave I was measured, mostly at 15 kHz, with offline software developed for research purposes, at the auditory phenotyping platform of the *Institut de l'Audition*. The amplitudes of ABR wave I in treated and non‐treated *Strc* ^−/−^ mice were normalised against the mean value of the ABR wave I from control wild‐type mice, which was set as 100%.

### 4.5 Distortion product‐otoacoustic emissions

We used an Otophylab (Echodia®) system to record distortion product otoacoustic emissions (DPOAE). The mice were anaesthetised with a mixture of ketamine and xylazine and placed on a heating pad, as described above. The DPOAE acoustic stimuli consisted of the simultaneous emission of two tones, *f*_1_ and *f*_2_, into the ear, with a ratio of *f*_2_/*f*_1 _= 1.2. The DPOAE response was measured at 2*f*_2_–*f*_1_ kHz. The *f*_2_ frequencies used were 12, 16, 20 and 32 kHz (6 and 8 kHz were excluded due to interference from background noise). The maximum intensity of *f*_2_ was limited to 70 dB, in accordance with the technical constraints of the Otophylab system, and intensity was gradually decreased, by 10 dB at a time, to 30 dB.

### 4.6 Cell transfection and Immunocytochemistry

HeLa cells were cultured in six‐well plates and transiently transfected with *Strc‐*NT, *Strc‐*CT or both plasmids using Lipofectamine 3000 (Thermo Fisher Scientific) according to the manufacturer's instructions. After 48 h, cells were fixed in 4% paraformaldehyde (PFA) diluted in phosphate‐buffered saline (PBS) solution for 1 h at RT, washed, and permeabilised in PBS containing 20% horse serum and.25% Triton X‐100 for 1 h. Cells were then incubated with an affinity‐purified polyclonal rabbit anti‐STRC antibody raised against the synthetic peptide EQLAYLSPEQRRAVA (amino acids 1753–1767 at the C‐terminus)^16^ (1:500 dilution) at RT for 1 h. After multiple washes cells were incubated for 1hr at RT with a goat anti‐rabbit antibody conjugated to Alexa Fluor 488 (1/500 dilution), DAPI (1/1000 dilution), and phalloidin (1/500 dilution). Finally, cells were rinsed with PBS and mounted on glass coverslips in Fluorsave® medium (Calbiochem, USA).

### 4.7 RT‐PCR

We employed reverse transcription PCR (RT‐PCR) to evaluate in vitro the recombination efficiency and accuracy of dual plasmids. To this end, human embryonic kidney (HEK 293) cells were cultured in six‐well plates and transiently transfected with either *Strc‐*NT or *Strc‐*CTor both plasmids using Lipofectamine 3000 (Thermo Fisher Scientific) according to the manufacturer's instructions. After 48 h, cells were lysed and total RNA was isolated using the RNeasy Kit (Qiagen). RT‐PCR was performed using the SuperScript™ III One‐Step RT‐PCR System (Thermofischer; 12574018) with *Strc*‐specific primers (forward: GTTCAACCATCCGAGGAGCA, reverse: CCTCCAGGGACAGATCATGC). For each RT‐PCR reaction, 1 µg of RNA extract was used as template. Sanger sequencing of PCR products was performed by Eurofins automated sequencing services.

### 4.8 Western blot

Transfected HEK 293 cells were transfected as previously described. Cell lysates were subjected to SDS–PAGE on 7.5% bis–tris acrylamide gels (Bio‐Rad) and then transferred onto polyvinylidene fluoride membranes. Immunoblotting was performed using standard protocols and the following antibodies: an affinity‐purified polyclonal rabbit anti‐STRC antibody raised against the synthetic peptide EQLAYLSPEQRRAVA (amino acids 1753–1767 at the C‐terminus)^16^ (1:1000 dilution) and an anti‐actin antibody (A3853; 1:2000 dilution; Sigma–Aldrich). Proteins of interest were detected with goat anti‐rabbit or anti‐mouse secondary antibodies coupled to horseradish peroxidase (1:5000 dilution; Jackson ImmunoResearch) and visualised using electrochemiluminescence (ChemiDoc MP Imaging System; Bio‐Rad).

### 4.9 Immunofluorescence microscopy

Mice were euthanised and their inner ears were excised and fixed by incubation in 4% PFA in PBS for 45 min at room temperature and then decalcified by incubation overnight in ethylenediaminetetraacetic acid (EDTA;.5 M) at 4°C. The inner ears were washed several times in PBS (3 × 10 min), and the organs of Corti were then microdissected and incubated for 1 h at room temperature in PBS supplemented with Triton‐X100 (.03%) and horse serum (20%) for permeabilisation and saturation.

The samples were then incubated overnight at 4°C with an affinity‐purified polyclonal rabbit anti‐STRC antibody raised against the synthetic peptide EQLAYLSPEQRRAVA (amino acids 1753–1767 at the C‐terminus)^16^ (1:500 dilution). The samples were rinsed several times with PBS and incubated with secondary antibodies, including an ATTO‐488‐conjugated goat anti‐rabbit F(ab′)2 IgG fragment (1:500 dilution, catalog A11039; Thermo Fisher Scientific) and TRITC‐conjugated phalloidin (1:500). In some cases, we bathed samples in DAPI (1:5000) for 20 min at room temperature to visualise the nucleus of the hair cells. Finally, the samples were rinsed with PBS and mounted on glass coverslips in Fluorsave® medium (Calbiochem). We used a Zeiss LSM‐900 Airyscan confocal microscope for sample visualisation, with the 63X oil immersion objective for higher magnifications.

### 4.10 Scanning electron microscopy

The inner ears were removed, and a hole was made at the apex of the cochlea. The cochleas were fixed in 2.5% glutaraldehyde diluted in.1 M sodium cacodylate (Sigma–Aldrich) for 2 h at room temperature and decalcified by incubation overnight in EDTA (.35 M) in.75% glutaraldehyde at 4°C. After 2 h of post‐fixation in 4% PFA at room temperature, the cochleas were rinsed and the organs of Corti were microdissected. The specimens were rinsed in.1 M cacodylate and subjected to an OTOTO protocol consisting of six alternative incubations in 1% tetraoxide osmium diluted in.1 M cacodylate for 1 h and.1 M thiocarbohydrazide for 15 min, with a wash in ultra‐pure water between incubations. The samples were then dehydrated in a graded series of ethanol solutions from 35 to 100% (35, 50, 70, 85, 95 and 100% ethanol) and dried to critical point by 15 min of incubation in hexamethyldisilazane. Finally, the specimens were mounted on carbon‐coated discs attached to aluminium stubs (Oxford Instruments) and covered with gold/palladium with a Quorum Q150R S sputter coater (Quorum Technologies). A JEOL JSM6700F electron microscope operating at 7 kV was used to obtain scanning electron images.

### 4.11 Surgery for intrinsic imaging and auditory behavioural tasks

During the 5th and 8th post‐natal weeks, we implanted a headplate in the mice to make it possible to maintain them in head‐fixed setup for both auditory behavioural tasks. Surgery was performed under general anaesthesia, as described above. The mice also received a dose of buprenorphine (Vétergesic;.05–.1 mg/kg) 30 min before surgery and a local dose of lidocaine (Laocaïne; 5 mg/kg) under the skin of the dorsal part of the skull 10 min before surgery. The metal head plate was affixed to the skull using dental acrylic (Ortho‐Jet, Lang). Following surgery, mice received oral meloxicam (1 mg/kg) administered in the drinking water and were monitored daily for five consecutive days prior to the initiation of behavioural testing.

### 4.12 Behavioural setup

Behavioural experiments were carried out following previously established procedures.^40^ Task control and data acquisition were implemented using custom MATLAB software (MathWorks) interfaced with a National Instruments PCIe‐6351 data acquisition board.

Auditory stimuli were amplified using a stereo power amplifier (SA1; Tucker‐Davis Technologies) and presented through high‐frequency speakers (MF1‐S; Tucker‐Davis Technologies) in a pseudo‐randomised order. Animals were positioned within a custom 3D‐printed restraining tube, and head fixation was maintained throughout the session.

Water rewards (5 µL each) were delivered via a solenoid valve (LVM10R1‐6B‐1‐Q; SMC).

Licking activity was monitored by applying a 5 V potential across a circuit connecting the lick spout and the aluminium platform under the mouse. Voltage deflections measured across a series resistor were used to detect lick contacts.

### 4.13 Behavioural procedures

*Sounds*: Auditory stimuli consisted of 500‐ms sound bursts delivered at 70 dB SPL and sampled at 192 kHz. The assignment of Go and No‐Go tones was counterbalanced between animals. During the frequency discrimination phase, mice learned to differentiate a low‐frequency tone (6 kHz) from a high‐frequency tone (16 kHz).

For the psychometric frequency discrimination test, two tone sets were used: eight low‐frequency tones (6–9.5 kHz) and eight high‐frequency tones (10–16 kHz), distributed evenly on a logarithmic frequency scale.

*Training protocol*: Before behavioural testing, animals were gradually habituated to the head‐fixation setup, starting with 15‐min sessions and progressively extending to 2 h.

Following habituation, a water‐restriction schedule was introduced. Mice received.8 mL of water per day, with free access to water overnight every Friday (16 h). Food was available ad libitum throughout the experiment, and body weight was monitored daily. Behavioural sessions were conducted 5 days per week (Monday–Friday).

Training was performed in successive stages: *Free‐lick phase*: On the first day, mice obtained water by licking the spout in the absence of auditory stimuli. *Go training*: Go trials were presented with a probability of 80%, interleaved with 20% blank trials. Each trial included a random inter‐trial interval (0–3 s), a no‐lick period (1.5–3.5 s) and a fixed 1.5‐s response window. Four consecutive licks during the response window were scored as a *hit* and immediately rewarded with water. Trials with fewer licks were considered *misses*. To maintain motivation, the first 10 trials of each session included automatic water rewards. *Go/No‐Go training*: After mice achieved >80% hit rate, a No‐Go tone was introduced. The lick‐count threshold (5–8 licks) was adjusted individually for each mouse. During No‐Go trials, responses below threshold were scored as *correct rejections*, whereas responses above threshold were considered *false alarms* and followed by a random time‐out (5–7 s) without reward. Go and No‐Go trials each occurred with a probability of 40%, and 20% were blank trials.

Once mice performed the Go/No‐Go task with >80% accuracy, they proceeded to the psychometric testing phase, which included 16 tones (8 low and 8 high frequencies) to generate psychometric functions for frequency discrimination. Each session continued until the animal had received a maximum of.8 mL of water or stopped responding to the stimuli (typically after 300–600 trials). If the full volume was not reached during the session, the remaining amount was provided in the home cage.

### 4.14 Statistics

All statistical analyses were carried out with Prism 9 (GraphPad, USA). We performed normality and lognormality tests to check that the data were normally distributed an ANOVA test was used to compare two or more experimental groups for larger datasets (*n *> 5) for normally distributed data. All values are reported as the mean percentage ± standard error of the mean. Differences were considered significant if *p* value < .05. Statistical significance is indicated in the figures as follows: ns., not significant; *p < .05; **p < .01; ***p < .001; ****p < .0001.

## AUTHOR CONTRIBUTIONS

S.I. and S.B. performed and analysed the experiments and wrote and revised the manuscript. C.F., M.C. and M.G. performed the in vitro experiments. H.L.R. contributed to behavioural task data acquisition. V.M. performed SEM image acquisition. S.J. and A.S. contributed to supplementary experimentations. M.J.L. and N.B. analysed data and revised the manuscript. S.S. and B.B. designed the study and revised the manuscript. S.S. wrote the manuscript. All authors agreed to the submission of the final version.

## CONFLICT OF INTEREST STATEMENT

The authors declare no conflicts of interest.

## ETHICS STATEMENT

Animal experiments were conducted in compliance with French and European regulations for the care and use of laboratory animals (EU Directive 2010/63 and French Law 2013–118, 6, February 2013), and were approved by the Institut Pasteur's Ethics Committee for Animal Experimentation.

## Supporting information



Supporting information

## Data Availability

All data that support the findings in this paper are available from SS upon reasonable request.
