# Supplementary material for: Dual AAV gene therapy achieves recovery of hearing and auditory processing in a DFNB16 mouse model
Source: Clin Transl Med. 2026 Jan 9;16(1):e70571. doi: 10.1002/ctm2.70571 (PMC12784207; doi:10.1002/ctm2.70571)
Supplement: Supplementary file 1 — Supporting information [file CTM2-16-e70571-s001.docx]

**Dual AAV gene therapy achieves recovery of hearing and auditory processing in a DFNB16 mouse model**

**Supplementary Figures**

**Supplementary figure S1. Dual AAV vector design**

**
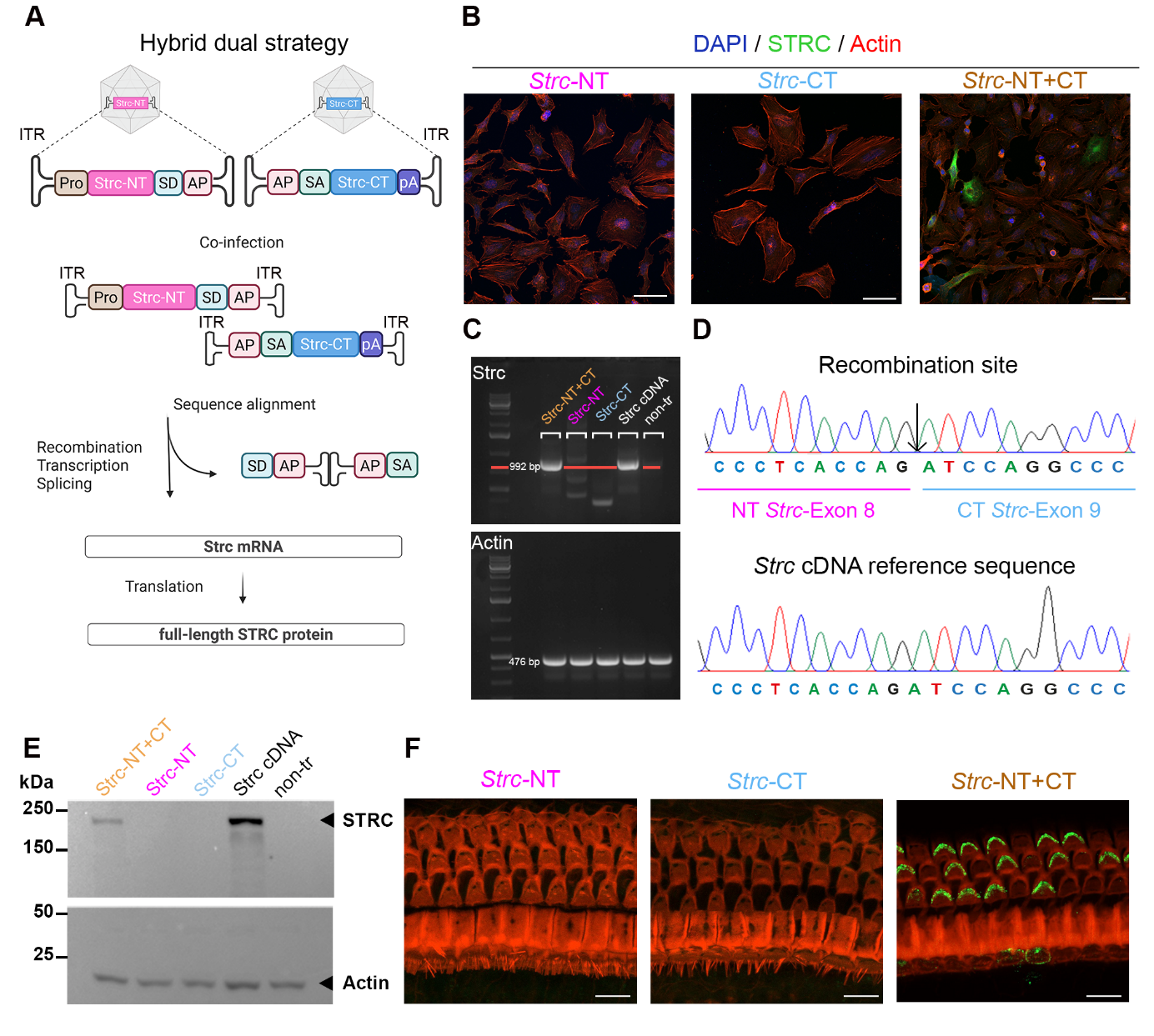
**

**(A)** Schematic representation of the hybrid dual AAV strategy used to deliver the fragmented murine *Strc* cDNA in this study. ITR, inverted terminal repeats; pA, polyadenylation signal; SA, splice acceptor site; SD, splice donor site*;* AP, recombinogenic alkaline phosphatase sequence, and Pro, promoter. **(B)** HeLa cells transfected with *Strc*-NT plasmid (left), *Strc*-CT plasmid (middle) and cotransfected with both plasmids (right), stained for STRC (green), DAPI (blue) for the nucleus, and phalloidin (red) for actin. STRC is detected exclusively in cells cotransfected with both the *Strc*-NT and *Strc*-CT plasmids. Scale bars, 50 µm. **(C)** RT-PCR targeting the *Strc* recombination region (upper panel) in transfected cells with normalization against actin (lower panel). Amplicons obtained from cells co-transfected with the *Strc*-NT and *Strc-*CT plasmids were of the expected size and identical in length to those amplified from *Strc* cDNA transfection. **(D)** Sanger sequencing of the *Strc* transcript encompassing the recombination region between the *Strc*-NT and *Strc*-CT fragments (upper panel) relative to the native *Strc* cDNA sequence (lower panel). **(E)** Western blot analysis of transfected cell extracts confirmed STRC protein expression exclusively in cells co-transfected with both plasmids (*Strc*-NT+*Strc-*CT), with a band at the expected molecular weight identical to that observed in extracts from cells transfected with full-length *Strc* cDNA. No signal was detected in cells transfected with either *Strc*-NT or *Strc*-CT alone, or in untransfected controls (Non-tr stands for non-transfected cells). Normalized against actin protein **(F)** Confocal image of the basal turn of the organ of Corti from *Strc*^-/-^ mouse cochleas treated with AAV-*Strc*-NT (left), AAV-*Strc*-CT (middle), or both AAV-*Strc*-NT + AAV-*Strc*-CT (right), immunostained for STRC (green) and actin (red), showing that stereocilin is expressed only in the cochlea into which both AAV vectors were injected. Scale bars, 10 µm.

**Supplementary figure S2. STRC re-expression rescues the structure of OHC bundles**

**
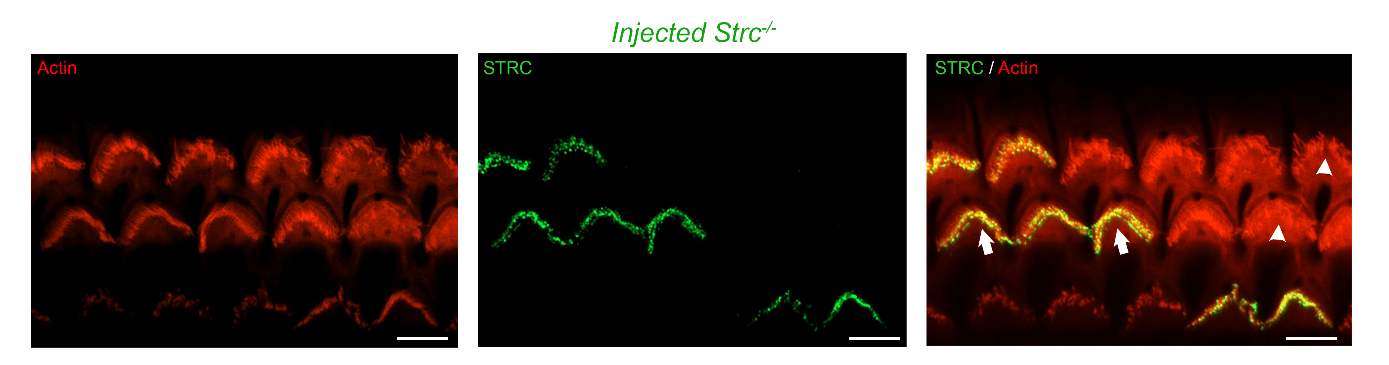
**

High-resolution Airyscan images of the organ of Corti from the basal region of injected Strc^-/-^ cochlea immunostained at P30 for STRC (green) and actin (red). Transduced OHCs exhibit normal stereociliary bundles with preserved V-shaped (arrows), whereas non-transduced OHCs lacking STRC protein displayed disorganized OHC bundles (arrowhead) Scale bars, 5 µm.

**Supplementary figure S3. Dual AAV gene therapy restores a sustainable auditory function in *Strc*^-/-^ mice.**


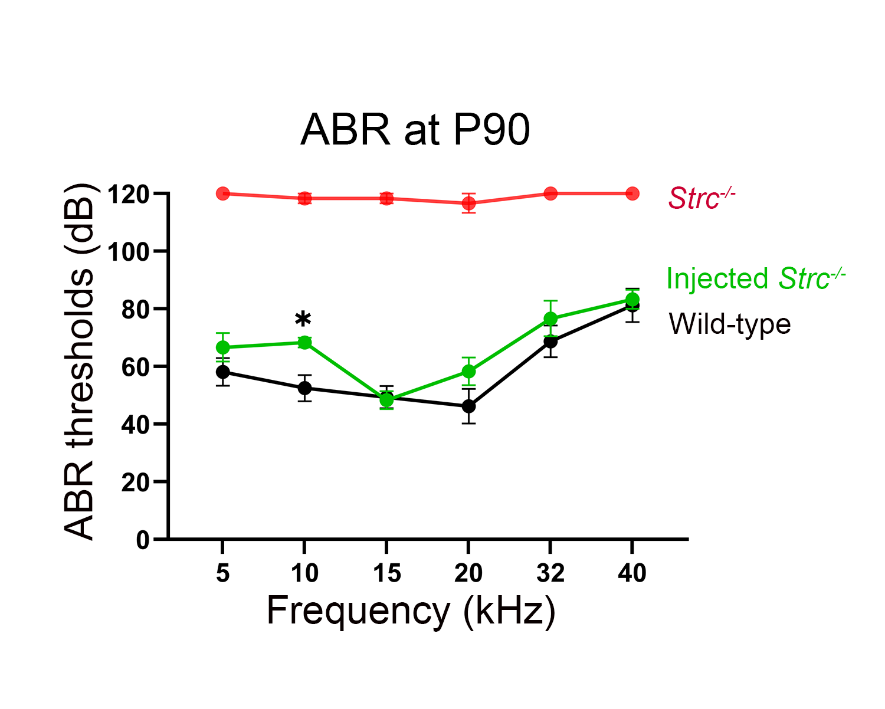


The ABR thresholds were recorded at P90 in untreated *Strc^-/-^* (red), treated *Strc^-/-^* (green), and wild-type (black) mice. Note that treated *Strc^-/-^* mice show ABR thresholds to near wild-type levels, with slight but significant elevation at 10 kHz. Data presents mean±SEM, *n*=6 per group, **p* < 0.05, Two-way ANOVA.

**Supplementary figure S4. Structure and protocol of the behavioral discrimination task.**


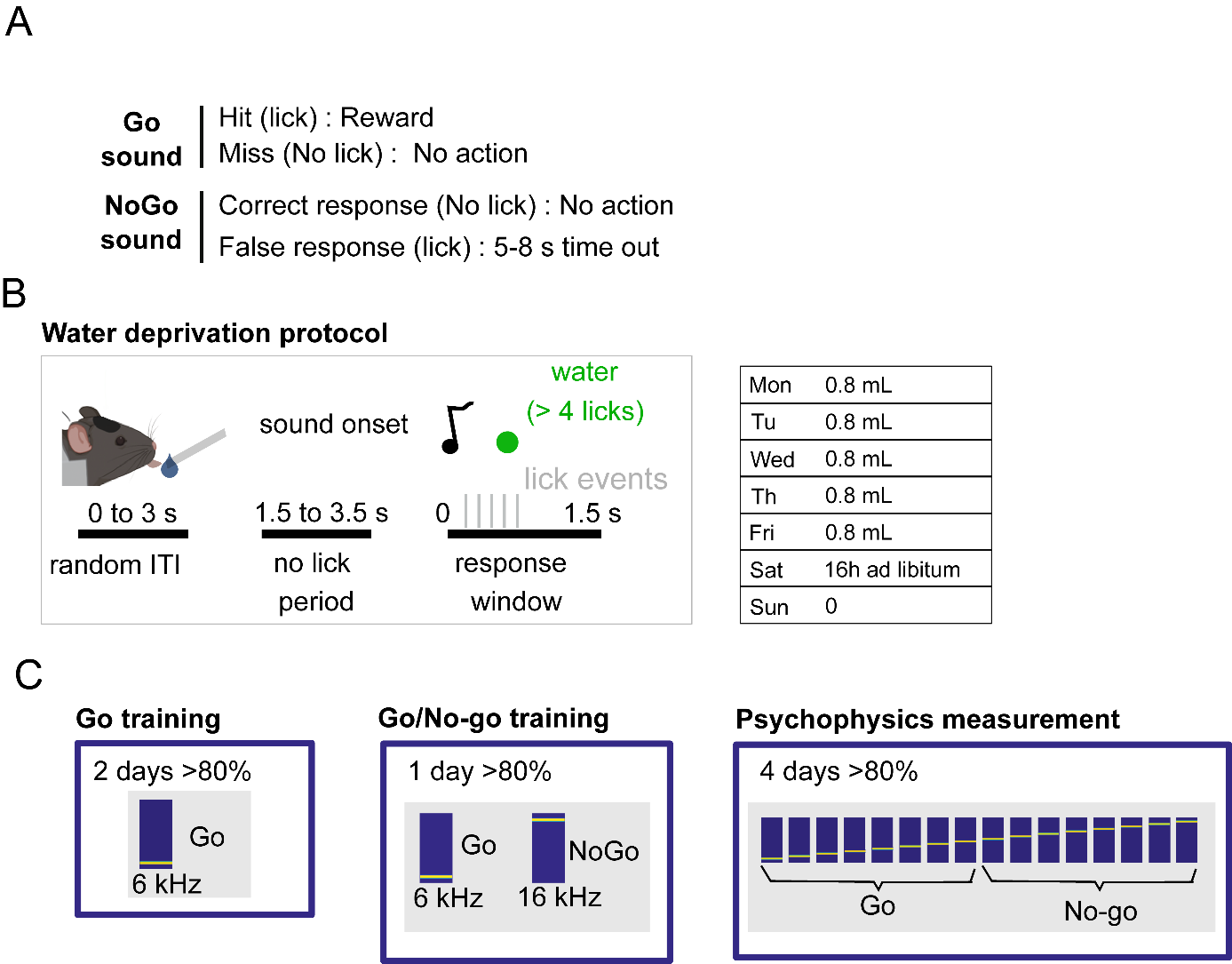


(A) Schematic representation of the Go/No-Go auditory task, illustrating the sequence of sound presentations and the corresponding behavioral outcomes leading to reward delivery.
(B) Outline of the water restriction schedule. Each trial included a randomized inter-trial interval (0–3 s) to prevent temporal prediction of the sound, a variable no-lick phase (1.5–3.5 s), and a fixed 1.5 s response window. A licking response exceeding the defined threshold (3–5 consecutive licks) during a Go trial was counted as a *hit* and triggered water reward. The right panel shows the daily water intake during training.
(C) Summary of the successive training stages and the accuracy criteria required for progression to the next phase of the task.
